# Should older people ever be discharged from hospital at night?

**DOI:** 10.1007/s11673-022-10194-8

**Published:** 2022-06-17

**Authors:** Brent Hyslop

**Affiliations:** Department of Medicine, Dunedin School of Medicine, University of Otago, Private Bag 1921, Dunedin, 9054 New Zealand

**Keywords:** Patient discharge, Aged, Vulnerable populations, Safety, Personal autonomy, Risk

## Abstract

The discharge of older people from hospital at night is a topical and emotive issue that has recently gained media attention in New Zealand and the United Kingdom, including calls to prevent it occurring. With growing pressures on hospital capacity and ageing populations, normative aspects of hospital discharge are increasingly relevant. This paper therefore addresses the question: Should older people (say, over eighty years old) ever be discharged home from hospital during the night? Or given safety concerns, should regulation against the night-time discharge of older people be put in place? Employing a principlist lens to bioethics, this paper considers key principles or values involved, including discharge safety concerns, personal preference and consent, the risk of remaining in hospital, and broader considerations around discharge policy. These points act as a possible framework for further research and discussion of normative aspects of hospital discharge. Overall, this paper argues that while discharge safety concerns must be properly acknowledged and addressed, it can still sometimes be appropriate for an older person to leave hospital at night. The option of night-time discharge should therefore remain open to people of all ages.

## Introduction

The discharge of older people from hospital during the night, including from emergency departments, assessment units and inpatient wards, is a topical and emotive issue. The issue has previously received significant political and media attention in the United Kingdom, including calls from politicians to stop the practice (Gregory 2016; Cooper 2014). Night-time discharge has now also gained national attention in New Zealand. Figures released showed that, in January 2021, 156 people aged over eighty years were discharged from hospital between 1 a.m. and 8 a.m. across New Zealand (Satherley 2021).^1^ In ensuing media reports, comments from public figures criticizing night-time discharge included: “I am completely and utterly disgusted that this is happening” and “Someone will die” (Hudson 2021a); “I can’t think of one good reason to be discharging a person over 80 from hospital in the early morning” (Northland Age 2021); and that, if elected to government, one political party would “make it very clear [that] it is unacceptable to discharge older people in these hours” (Satherley 2021). A government minister added that they would be “absolutely mortified” if their own grandmother was discharged in the middle of the night (Satherley 2021).

Given its importance, increasing frequency, and potential policy implications, the issue of night-time discharge has received relatively little attention outside of popular media (Roseveare 2014; Lees and Dyer 2012). This paper therefore addresses the question: Should older people (say, over eighty years old) ever be discharged home from hospital at night?^2^ Or given safety concerns and criticism, should some form of regulation against the practice be put in place, with discharge only occurring the following day? Employing a principlist lens to bioethics, this paper considers the broad principles or values involved in the issue, as a possible framework for further research and discussion. It adds to a growing body of literature on normative aspects of hospital discharge (O’Keeffe 2015; Mukherjee 2015; Hyslop 2020), which given current pressures on hospital capacity and ageing populations is an increasingly important area.

As in the media, this paper focuses on older people, but the analysis could also apply to night-time discharge in relation to other population groups, such as those in remote geographical settings (Fellner 2021). Although the exact setting of hospital discharge varies, including discharge from emergency departments, assessment units and inpatient wards, the underlying principles are similar: Should this person, at this time, remain in hospital or return home? This paper therefore uses the term “hospital discharge” to include all settings. This paper also assumes that, when discharge at night is considered, people have been deemed medically fit for discharge (that is, there is no diagnostic or therapeutic reason why they need to stay in hospital) and that the issue is instead about a person’s function and ability to practically manage a return home at night.

## Discharge Safety Concerns

In addressing night-time discharge, several points or principles should be considered. A first, widely accepted point, as emphasized in popular media, is that hospital discharge at night comes with a greater risk of adverse events and harm than discharge during the day, and therefore raises safety concerns. Reports like a man being found at night by police “crying, disorientated, confused” and “blue with cold” on a sports field an hour after hospital discharge are obviously alarming and need to be addressed (Cooper 2014). People with reduced physiological reserve (or “frailty”), cognitive impairment, and/or functional limitation can struggle with routine tasks necessary for a successful return home from hospital, such as organizing transport and medication, accessing and heating their home, and managing basic activities of daily living such as toileting and food preparation. Older people have higher rates of these conditions and are therefore as a group more susceptible to having difficulty managing and to suffering harm after hospital discharge (Hestevik et al. 2019; D’souza and Richards 2018). When discharge occurs at night, there will generally be less support services and physical care immediately available in a person’s home. Night-time discharge will therefore be inherently challenging for many older people.

In addition to these inherent challenges, there is also concern around the quality of planning and care provision in a person’s return home from hospital—an area referred to as discharge planning. Unfortunately (and unacceptably), cases of inadequate discharge planning occur and are now well documented (Parliamentary and Health Service Ombudsman 2016). Recognized shortcomings in discharge planning include insufficient practical support being provided for people returning home and poor communication between hospitals and community care providers. Inadequate discharge planning can result in avoidable harm to older people and distress to them and their families (Parliamentary and Health Services Ombudsman 2016). This is again of increased concern at night when people’s support networks are generally less available and when delays in communication will sometimes be inevitable. Given that hospitals typically have fewer staff working overnight, there might also be relatively less staff resource available to optimally facilitate discharge. And when a patient’s medication has been added to or changed, the availability of medicines and pharmacy services is another significant issue at night (Satherley 2021). This concern about night-time discharge planning has been specifically documented by a recent report that found that twenty-seven per cent of adults of all ages who were discharged at night felt unprepared to return home (Healthwatch England and British Red Cross 2020). So given both the inherent challenge of discharge at night for many older people and the need for adequately thorough discharge planning, there is valid and significant concern about the safety of hospital discharge during the night, as highlighted by media reports.

An additional, related concern is that in some cases an older person might not be able or comfortable to enunciate or explain reasons why they shouldn’t be discharged at night, so that “it’s possible they could be taken advantage of” (Satherley 2021). This is of particular relevance during periods of high hospital demand and limited bed availability, when there could be pressure on staff to discharge current patients to free up beds for other people requiring urgent hospital care. There is also a background of general concern that older people are often not adequately involved in decision-making about their care (Holmes and Ibrahim 2021). These factors could therefore result in people being discharged home at night to situations in which it is difficult for them to manage and/or when they would prefer to stay in hospital until morning. While it is maintained that there is no general right to remain in hospital when doing so is not clinically indicated (Swidler, Seastrum, and Shelton 2007), it is essential that the challenge of returning home at night, and the potential for significant harm and distress, are properly considered and addressed. It might even be argued that hospitals should (and do) fulfil a role in the care and support of society beyond that of providing medical care. In either case, a duty to protect potentially vulnerable people—whether medically or socially vulnerable—needs to be recognized. It is therefore also essential that staff ensure that an older person’s thoughts and preference about possible discharge at night are genuinely sought and respected, realizing that they will generally have the best understanding about their own unique situation.

## A Person’s Discharge Preference

But despite potential or actual difficulties in returning home, a person might in some cases genuinely prefer to discharge at night rather than staying in hospital until morning. For instance, a person’s over-riding priority might be to return to the familiar comfort of their own home (and likely to a quieter and better night’s sleep), rather than staying overnight as a dependant in a public institution. They might still wish to discharge at night even when there is significant risk and concern about their ability to manage at home. How then should staff and hospitals respond to such a situation, where a person’s preference potentially conflicts with a duty of protecting vulnerable people? As has been suggested in media reports, is a prohibition on night-time discharge for older people appropriate for their safety and welfare?

Scenarios where a person’s preference appears to conflict with their welfare have been extensively considered in health ethics and law. A second broad principle in relation to night-time discharge follows from this. It is now well-established in the common law that an adult person’s autonomous choice about whether or not to receive a healthcare intervention takes primacy over considerations of their health and welfare. This principle applies even where a person’s choice could result in serious harm or death. As stated in the judgment of the Australian case *Hunter and New England Area Health Service v A* [2009] NSWSC 761, “whenever there is a conflict between a capable adult’s exercise of the right to self-determination and the State’s interest in preserving life, the right of the individual must prevail.” In New Zealand, this right to self-determination in healthcare settings is specifically protected by the Code of Patients’ Rights, Right 7(1): “Services may be provided to a consumer only if that consumer makes an informed choice and gives informed consent” (with specified exceptions) (Health and Disability Commissioner (Code of Health and Disability Services Consumers’ Rights) Regulations 1996). The New Zealand Bill of Rights Act 1990, section 11, also provides a right to refuse medical treatment. These provisions show the high value placed on respect for a person’s autonomous choice and freedom from interference in healthcare, which clearly extends to decisions about whether or not to stay in hospital.

It follows then that a person should not be kept in hospital without their valid consent for doing so, unless a specific legal exception exists (for example, under mental health compulsory admission legislation). To do so could attract various types of legal and disciplinary liability (Skegg 2015). In the case of an older person wishing to leave hospital at night, if they retain decision-making capacity and are acting voluntarily with adequate information (that is, if their refusal of continued hospital admission is valid), then it is appropriate that their discharge is facilitated in a timely manner, even if safety concerns exist. The introduction of a blanket rule of “no night-time discharge for older people” could therefore be criticized as being overly paternalistic, possibly leading to the infringement of well-established and fundamental rights.

Respecting an older person’s preference to discharge at night does not mean, however, that safety should be overlooked. On the contrary, staff should always recognize risk and engage collaboratively with patients and their support networks to optimize safety and welfare as much as the circumstances allow. Firstly, high quality, anticipatory discharge planning and coordination of care remains essential at night, given the concerns mentioned in the previous section (“Discharge Safety Concerns”). In particular, it is vital that a person is correctly informed about what support will be available for them at home, when this is able to be provided, and what to do if they have further problems. Secondly, family and caregiver views should be sought when relevant and taken into consideration, particularly when they are providing practical support and when there are significant concerns about discharge. However, situations can arise where staff might become preoccupied with the opinions of family members, rather than supporting an older person’s central role in decision-making about their own care (Holmes and Ibrahim 2021). It needs to be remembered that legal consent or refusal for healthcare comes from the patient, not from family members (with specific exceptions in some cases, such as guardianship legislation). Thirdly, in some cases a person might lack capacity to decide about whether to remain in hospital. If so, then a decision by an authorized substitute decision-maker or a “best interests” decision might be necessary, following local legal processes. Whether or not a person retains capacity to decide about their potential discharge is therefore a vital (and sometimes complex) consideration, triggering different approaches in discharge planning. In any case, the aim should be open, collaborative, and person-centred discussion and planning, recognizing and addressing safety concerns that exist, while still respecting an older person and their preference (Hyslop 2020). To achieve this, an option of night-time discharge should remain available to people of all ages.

## The Risk of Remaining in Hospital

In addition to discharge safety concerns and respect for a person’s preference, a third point is relevant in relation to night-time discharge: the risk of remaining in hospital overnight. This point has received little attention in media commentary. While hospital admission is by its nature considered necessary or beneficial, time in hospital also carries significant risk for individual patients (Hauck and Zhao 2011). For older people, complications such as delirium, falls and fractures, pressure sores, incontinence, and loss of function are of particular concern while in hospital (Mudge and Hubbard 2019), in addition to the general risk of adverse events. Recently, a concept of hospital admission risk or “not safe for admission” has begun to emerge (Hullick, McNamara, and Ellis 2021). While a person might have some reason for admission to hospital, it could be possible that their risk of complications in hospital (due to their pre-existing comorbidities and functional issues) outweighs the potential benefit of admission.

Consider, for example, an older man with visual impairment presenting to hospital in the evening after a brief episode of now resolved chest pain, subsequently diagnosed as muscular pain with no significant pathology and no need for additional care. If adequate support is available to properly facilitate discharge, his return home at night doesn’t appear to introduce new risks. However, remaining in hospital overnight does introduce new risks, such as risk of falling and injury in an unfamiliar environment, hospital-acquired infection, and poor sleep and its consequences. In practice, such straightforward cases like this example are not typical, and predicting risks of admission and discharge is difficult. Older people often might present with multiple and/or non-specific health and functional issues, which complicates risk assessment. Regardless, the point here is to recognize that a balanced analysis of night-time discharge requires not only consideration of risks of discharge, but also of risks of admission. As said: “If we are concerned about the safety of discharge home, should we not also be concerned about the safety of hospital admission? Hospitals are dangerous places, particularly for older people and those with chronic illness” (Goodacre 2006).

Sound clinical decision-making requires staff to consider both the potential benefit and the risks of each available management option or alternative. In this context, this includes both the potential benefit and risk of remaining in hospital overnight and the potential benefit and risk of discharge home. Patient-centred care also requires that potential benefits and risks are considered from each patient’s own perspective. In some cases, this potential benefit/harm balance will favour discharge home, even during the night, and so it should be appreciated that overnight hospital admission is “not always in [an older person’s] best interests, as there are other potential risks that can be introduced during an inpatient hospital stay” (Hudson 2021b). While it must be recognized that discharge at night raises safety concerns, remaining in hospital can also raise additional safety concerns (and potential discomfort), and this point should be considered in addressing the issue of night-time discharge.

## Broader Considerations

This paper has mainly focused on night-time discharge decisions in relation to the individual patient. There are also broader reasons why a rule against night-time discharge for older people might have “unintended consequences” (Gregory 2016). Currently, it is maintained by clinicians and hospitals that many night-time discharges are appropriate and supported by patients and their families (Hudson 2021b, Roseveare 2014). To delay these appropriate discharges would be to then occupy hospital beds unnecessarily, potentially delaying care for other patients. Obviously, inappropriate discharges should be avoided, and staff must ensure that all night-time discharges are in keeping with patients’ wishes and are adequately supported and planned and that people are never discharged just to relieve hospital capacity pressure. But to keep people in hospital for a longer period just because of their age, when they don’t need or want to be there, would be an inefficient use of scarce and valuable resource. On the other hand, a further potential concern is that the introduction of a requirement to stay in emergency departments or assessment units until a night is over might possibly be regarded by some older people as a major discomfort and/or loss of control or dignity. If so, this could lead to (or add to) a reluctance by some people to attend a hospital for healthcare when needed, especially when this need arises later in the day or at night. Therefore, it is possible that policy restricting night-time discharge could adversely interfere with appropriate hospital access and patient flow, both into and out of hospitals.

Furthermore, an age-based discharge restriction could unintentionally contribute to stereotypes and negative attitudes toward older people. This could occur in two ways: firstly, by grouping all older people together without appreciating the huge biological, psychological, and social variation that exists between older individuals; and secondly, by emphasizing “vulnerability” instead of positive aspects of ageing such as wisdom and experience (Falconer and O’Neill 2007). There is already concern that older people may be excluded from decision-making about their own care due to ageist attitudes and a culture of paternalism towards older people (Holmes and Ibrahim 2021). A rule against night-time discharge would reduce older people’s options and control in this setting, which could further contribute to negative stereotypes and perspectives.

What is needed, then, is an understanding and consideration of each person and their case in its context, rather than a blanket rule on hospital discharge based on discharge risk and chronological age alone (Lees and Dyer 2012). As in healthcare decision-making in general, decisions about older people and discharge at night should be person-centred, recognizing that each person has their own unique circumstances, values, and priorities in life. And as with hospital discharge in any setting, decision-making and planning should be open, collaborative, and respectful of each patient’s preference. Given the significant concern about the safety of night-time discharge for older people, high quality, thoughtful discharge planning is vital and careful attention should be paid to the issue. However, key principles and values involved—personal preference and autonomy, balanced risk-benefit analysis, appropriate resource use, and a positive perspective on ageing—support the option of night-time discharge from hospital remaining open and available to people of all ages.

## Footnotes

1 In New Zealand, an emergency department stay of over three hours is counted as an admission with subsequent discharge. Numbers of discharges by hospital location were not provided.

2 An arbitrary age of eighty years old is mentioned to help focus this analysis, while recognizing the importance of “biological age” and function in this context, as well as “chronological age” (Wu et al. 2021).
